# A Qualitative Analysis of Cultured Adventitious Ginseng Root’s Chemical Composition and Immunomodulatory Effects

**DOI:** 10.3390/molecules29010111

**Published:** 2023-12-23

**Authors:** Hong Chen, Xiangzhu Li, Hang Chi, Zhuo Li, Cuizhu Wang, Qianyun Wang, Hao Feng, Pingya Li

**Affiliations:** 1 School of Mechanical Engineering, Shanghai Jiao Tong University, Shanghai 200240, China; chenhong@thdb.com; 2 Tonghua Herbal Biotechnology, Co., Ltd., Tonghua 134123, China; lixiangzhu@thdb.com (X.L.); chihang@thdb.com (H.C.); 3 School of Pharmaceutical Sciences, Jilin University, Changchun 130021, China; lizh0205@jlu.edu.cn (Z.L.); wangcuizhu@jlu.edu.cn (C.W.); wqy22@mails.jlu.edu.cn (Q.W.); 4 College of Basic Medicine Sciences, Jilin University, Changchun 130021, China; haofeng@jlu.edu.cn

**Keywords:** cultured adventitious root of ginseng, ginsenosides, UPLC-QTOF-MS, immunomodulatory, network pharmacology analysis

## Abstract

The cultivation of ginseng in fields is time-consuming and labor-intensive. Thus, culturing adventitious ginseng root in vitro constitutes an effective approach to accumulating ginsenosides. In this study, we employed UPLC-QTOF-MS to analyze the composition of the cultured adventitious root (cAR) of ginseng, identifying 60 chemical ingredients. We also investigated the immunomodulatory effect of cAR extract using various mouse models. The results demonstrated that the cAR extract showed significant activity in enhancing the immune response in mice. The mechanism underlying the immunomodulatory effect of cAR was analyzed through network pharmacology analysis, revealing potential ‘key protein targets’, namely TNF, AKT1, IL-6, VEGFA, and IL-1β, affected by potential ‘key components’, namely the ginsenosides PPT, F1, Rh2, CK, and 20(*S*)-Rg3. The signaling pathways PI3K–Akt, AGE–RAGE, and MAPK may play a vital role in this process.

## 1. Introduction

Ginseng (Panax ginseng C. A. Meyer), known as ‘the King of Herbs’, has demonstrated biological activities in heart protection [1,2,3], as well as anti-tumor [4,5,6], anti-inflammation [7,8], and anti-oxidation [9,10] activities, among other benefits. The root of ginseng contains the majority of its saponins, which are the predominant active ingredients in ginseng. However, the cultivation of ginseng in fields usually requires 5–7 years before it can be harvested, and it is a labor-intensive process. The yield is highly susceptible to environmental factors, including climate, soil, pathogens, and pests, which limits the commercial usage of ginseng at a low cost. The in vitro culturing of ginseng adventitious root (AR) has shown great potential as an alternative method of producing ginsenosides [11].

Adventitious root culturing is an effective approach to the accumulation of ginseng biomass. It only takes weeks before the roots are ready for harvesting, and the culture conditions are highly controllable in a bioreactor [12]. Most importantly, it has been demonstrated that the composition of an adventitious root can be adjusted by modifying the ingredients of the culture media [13,14,15]. A study on the influence of temperature and light on the culture of hairy roots has reported an optimal condition of 20 °C/13 °C over a day (12 h)/night (8 h) cycle [13]. The production of ginsenosides can be significantly affected via the addition of methyl jasmonate in a ginseng–echinacea co-culture system [14]. Similarly, organic germanium can also improve the biomass and accumulation of ginsenosides in cultured adventitious roots (cARs) [15]. Thus, it is practical to evaluate the chemical compositions of ginseng cultured from a specific culturing protocol. Recently, we established an optimized cAR protocol that has been shown to achieve higher biomass and production in large-scale bioreactors. In this study, we employed UPLC-QTOF-MS coupled with UNIFI to analyze the ingredients of ginseng cAR. UPLC-QTOF-MS is a fast and accurate technique with high sensitivity that generates mass spectrometric fragmentation signals for the simultaneous determination of multiple components. It has been widely used in analyzing the chemical ingredients of ginseng root, leaf, berry, stem, etc. [16,17]. The UNIFI Scientific Information System is an informatics platform with an embedded Traditional Medicine Library that enables the rapid, comprehensive, and accurate identification and analysis of ingredients in cAR. In this study, we analyzed components with molecular weights ranging from 100 to 1500 Da in ginseng cAR.

Ginseng has been widely reported as an immune system modulator [18,19,20]. Various parts of ginseng can maintain immune homeostasis and enhance the immune response to microbial attacks. There are various types of cells in the immune system, and they respond differently to ginseng treatment. Ginseng extract can enhance the phagocytic activity of macrophages [21,22], drive the maturation of dendritic cells [23], enhance natural killer cell functions [18,24,25], induce antigen-specific antibody responses [26,27], control proinflammatory cytokine responses [28,29,30], etc. However, there have been no studies on the immunomodulatory effect of cAR of ginseng. In this study, we evaluated how cAR extract influenced the immune system using multiple mouse models.

## 2. Results

### 2.1. The Total Saponin and Total Polysaccharide Content

For calculating the total saponin content, the regression equation was Y = 0.00627x − 0.00633 (r = 0.9996), and the linear range was 10 μg to 100 μg. The total saponin content of the cultured adventitious roots (cAR) was 11%.

For calculating the total polysaccharide content, the regression equation was Y = 4.5879X + 0.0523 (r = 0.9994), and the linear range was 0.02 mg to 0.16 mg. The total polysaccharide content of the cAR was 1.07 g/100 g.

### 2.2. Identification of Components from the Cultured Adventitious Root of Ginseng

**Figure 1:**
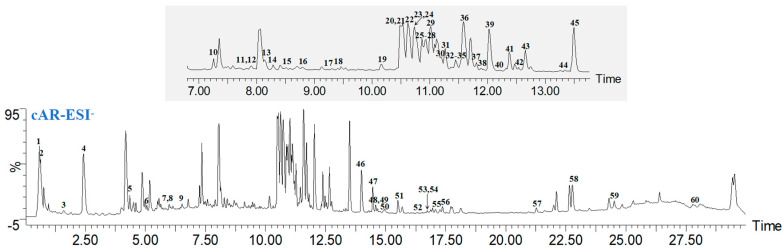
Representative BPI chromatograms of ginseng cultured adventitious root (cAR) extract in negative mode.

The components of ginseng cAR were identified using UPLC-Q/TOF-MS. The base peak intensity (BPI) chromatograms were measured in ESI^−^ modes (Figure 1). Through an analysis based on their mass, retention time (tR), and fragmentation, a total of 60 components were identified from cAR extracts in ESI^−^ modes, comprising 50 saponins, 1 steroid, 4 fatty acids, 3 phenolic acids, 1 amino acid, and 1 sugar (Table 1). Among the compounds, 32 were confirmed using chemical standards, namely sucrose, quinic acid, tryptophan, the notoginsenosides R1, Rg1, Re, Rf, F5, and Rb1, the notoginsenosides R2, Rb2, 20(*R*)-Rg2, 20(*S*)-Rh1, Ro, Rb3, Rs1, F1, Rc, Rd, and Rs2, the gypenosides XVII and Rd2, and the notoginsenosides Fd, 20(*S*)-Rg3, F4, 20(*R*)-Rg3, 20(*S*)-Protopanaxatriol, Rh2, CK, linolenic acid, linoleic acid, and 9-octadecenoic acid. In total, 24 components were putatively identified by comparing the tR and characteristic MS fragments with published results, and a total of four compounds were compared with CFM-ID 4.0. The structures of these compounds are shown in Figure S1.

### 2.3. Body Weights and the Organ/Body Weight Ratio

**Figure 2:**
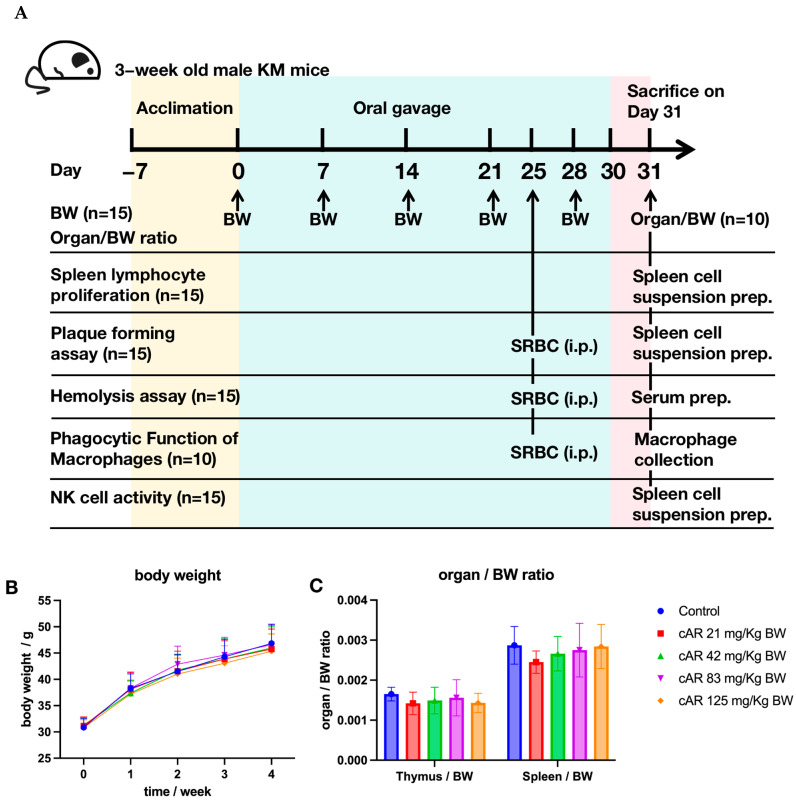
(**A**) Experimental design of the biological assays in this study. ‘n’ represents the number of animals in each group in a specific assay. (**B**) The influence of cAR extract on the average weekly body weight (BW) of the mice in each group (*n* = 15). (**C**) The thymus/BW and spleen/BW ratios of each experimental group (*n =* 10).

We administered cAR extracts to mice through oral gavage for 30 days (Figure 2A). The body weights of mice in all five groups, namely 0 (negative control), 21, 42, 83, and 125 mg/kg of body weight (BW) of cAR, were recorded once per week for four weeks (Figure 2B, Table S1). There was no significant body weight loss after the cAR treatment. The thymus/body weight ratio and the spleen/body weight ratio of the mice were also measured (Figure 2C, Table S1), showing no significant changes after the 30-day cAR treatment compared to the negative control. We also examined the appearance of the thymus and the spleen in each group. We did not observe obvious differences between the negative control group and the cAR treatment groups. Thus, we consider the maximum dose of cAR (125 mg/kg BW) to be non-toxic.

### 2.4. Spleen Lymphocyte Proliferation

**Figure 3:**
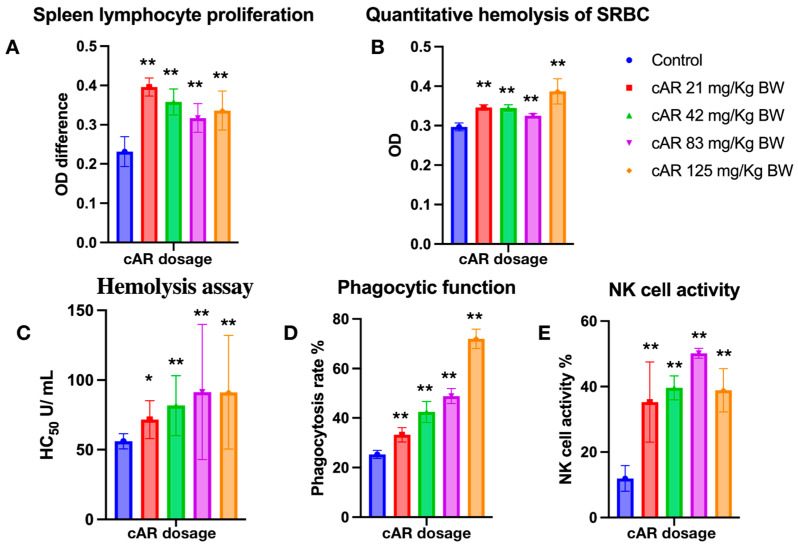
Evaluation of ginseng cAR extract in terms of enhancing the immune response during multiple biological tests in mice. (**A**) Spleen lymphocyte proliferation with MTT assay (*n* = 15); (**B**) quantitative hemolysis of SRBC (QHS) (*n =* 15); (**C**) hemolysis assay (*n =* 15); (**D**) the phagocytic function of the peritoneal macrophages (*n =* 10); (**E**) natural killer cell activity (*n =* 15). * *p* < 0.05 vs. the control and ** *p* < 0.01.

Lymphocytes and their subgroups are vital to the immune response process. They recognize pathogens, respond to eliminate antigenic substances, maintain the stability of the body’s environment, and protect the body. Lymphocytes can assist in cell−mediated immunity, indirectly boosting immune function. We assessed how cAR extracts can modulate cell-mediated immunity by measuring spleen lymphocyte proliferation. T lymphocytes require external stimulation to differentiate and expand from a resting state. Concanavalin A (Con A) is a mitogen that often serves as a substitute stimulant, rather than antigens. In T cell stimulation, Con A irreversibly binds to glycoproteins on the cell surface, inducing the proliferation of T lymphocytes. The MTT proliferation assay showed that T lymphocytes proliferated significantly after the administration of cAR (Table 2, Figure 3A). However, the lymphocyte proliferation did not occur in a dose−dependent manner.

### 2.5. Quantitative Hemolysis of SRBC (QHS) Assay

Humoral immunity is another aspect of immune function, and it refers to the formation of effector B cells and memory cells generated by B cells after stimulation via antigens. Effector B cells secrete antibodies to clear antigens, while long-lived memory cells are produced to continuously surveil the same antigen in the blood and lymph for future immune responses. The enhancement of humoral immunity can be evaluated through antibody formation or serum hemolysis. In this study, we employed a quantitative hemolysis of SRBC assay, measuring the optical densities at 413 nm. After the treatment with cAR, the formation of lymphoid cell antibodies significantly increased (Figure 3B, Table 2).

### 2.6. Hemolysis Assay

The hemolysis assay assesses the extent to which red blood cells (RBCs) are lysed by measuring the released hemoglobin in the surrounding fluid. In this study, sheep RBCs, as exogenous cells, were used for immunization in experimental mice. Thus, the immune cells of mice would recognize SRBC and lyse them. By measuring the OD of the released oxidized hemoglobin, the hemolysis reactions were assessed, and the immunomodulatory effects of cAR on humoral immunity were evaluated. In this study, all cAR treatment groups showed significantly increased HC_50_ values (Figure 3C, Table 2).

### 2.7. Phagocytic Function of Peritoneal Macrophages

The mononuclear–phagocyte system consists of mononuclear cells in the blood and macrophages in tissues, both of which have phagocytic functions to eliminate foreign substances. The mononuclear–phagocyte system can also secrete protective substances such as interleukin-1 (IL-1), interferons, and complement. Thus, the phagocytic function of macrophages is another indicator used to evaluate the immune function of the body. We employed the well-characterized method of the phagocytosis of chicken RBCs via mouse peritoneal macrophages to measure the phagocytic function in this study. Figure 3D shows that cAR can significantly increase the phagocytic function in mice in a dose-dependent manner.

### 2.8. Natural Killer Cell Activity

NK cells are another important type of immune cell in the body, and they are distinct from T and B cells. Instead of recognizing antigens, they distinguish abnormal tissue cells within the body from normal self-tissue cells. Activated NK cells can secrete various cytokines, regulate immune and hematopoietic functions, and directly kill target cells. Thus, an improvement in NK cell activity means an improvement in immune function. To assess the activity of NK cells after the cAR treatment, a lactate dehydrogenase (LDH) assay was used. Compared to the negative control group, all groups treated with cAR showed improved NK cell activity (Figure 3E, Table 2).

### 2.9. Network Pharmacology Analysis

**Figure 4:**
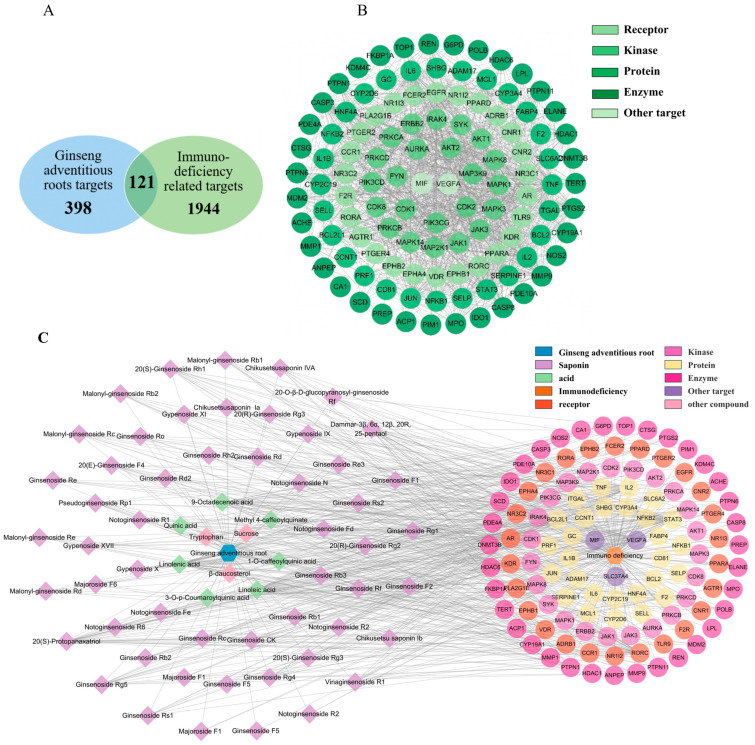
Results of the network pharmacological analysis. (**A**) Intersection proteins of cAR and immunodeficiency targets; (**B**) classification of intersection proteins; (**C**) cAR components–core targets network.

We have demonstrated that ginseng cAR performs immunomodulatory activities. In this section, we employed network pharmacology analysis to study the corresponding protein targets and related pathways of the biological activity of cAR. We queried multiple databases and identified 398 protein targets of the 60 components of cAR. In total, 1944 protein targets related to immunodeficiency were cross-compared with the cAR targets (Figure 4A). In total, 121 intersection proteins were considered potential targets responsible for the immunomodulatory effects of cAR. These intersection proteins were connected by 114 nodes and 1252 edges (Figure 4B). Of the intersection proteins, 38 were enzymes, constituting 31.4% of the intersection proteins. The rest comprised 26 receptors, 23 kinases, and 34 other proteins. The top five key targets, selected based on the degree value from the PPI network, were tumor necrosis factor (TNF), RAC-alpha serine/threonine-protein kinase (AKT1), interleukin-6 (IL-6), vascular endothelial growth factor A (VEGFA), and interleukin 1 beta (IL-8β).

Next, we constructed the ‘cAR components—core targets’ network to identify the potential key components of cAR extract corresponding to its immune-enhancing activity (Figure 4C). This network contained 180 nodes and 702 edges. Eighty-three percent of the components were ginsenosides, indicating the importance of ginsenosides. The top-10-ranking key components were selected based on their degree values, and they are listed in Table 3.

**Figure 5:**
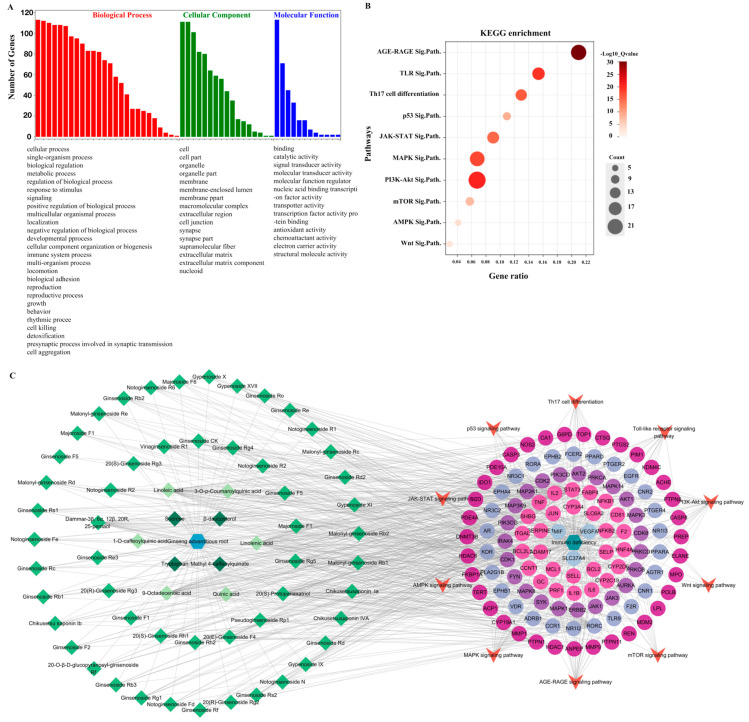
Results of the network pharmacological analysis. (**A**) GO enrichment, GO terms are in descending order; (**B**) bubble chart of KEGG enrichment (Sig.Path. represents signaling pathway); (**C**) cAR components–core targets–key pathway.

The core targets were also analyzed using GO enrichment and KEGG signaling pathway enrichment (Figure 5). The GO analysis revealed 55 GO entries comprising 26 biological processes, 17 cellular components, and 12 molecular functions (Figure 5A). We selected 10 of the 181 enriched KEGG pathways based on their *p*-values and published results (Figure 5B). The *p*-value is represented by color in the figure, while the number of genes related to the specific pathway is proportional to the size. Thus, the size and color of the bubbles illustrate the significance of these signaling pathways in the immunomodulatory activity of cAR. Three pathways, PI3K-Akt, AGE–RAGE, and MAPK, had larger bubbles with darker colors. As shown in Figure 5C, the ‘cAR components-core targets-key pathway’ network was established. This network had 190 nodes and 840 edges. The degree value of the PI3K-Akt, AGE–RAGE, and MAPK pathways was greater than that of others, which was consistent with the KEGG enrichment results. This further confirmed the significance of these three pathways in cAR activities.

## 3. Discussion

Chromatography-coupled mass spectroscopy methods have been widely used in studying the molecular compositions of different species of field-cultivated Panax ginseng. Researchers have discovered over 600 types of ginsenosides. However, to date, there has been no comprehensive assessment of the ingredients of cultured ginseng. Most studies have focused on evaluating the accumulation of ginsenosides using extraction and HPLC isolation, as described by Yu [46]. In this study, UPLC-QTOF-MS was employed to perform an unbiased screening of cAR components. We identified 60 components in the ESI^−^ mode, of which 32 were confirmed using chemical standards, 24 were putatively identified by comparing the retention times and characteristic MS fragments with published results, and 4 were compared with CFM-ID 4.0. It is worth mentioning that a quantitative study of cAR ingredients was begun in our lab. A comprehensive comparison of cAR and cultivated ginseng in fields will be performed as soon as we collect these data.

The immunomodulatory effects of field-cultivated ginseng have been extensively reported [18,21,47,48,49]. Kang and Min reviewed over a hundred published works before 2012 on the ‘immune boost’ activities of ginseng in relation to innate immunity, acquired immunity, and cytokines [47]. Kim recently reviewed the immunomodulatory effects of different types of ginseng, including white ginseng, red ginseng, and individual ginsenosides [48]. Although there are no published results on how cAR influences the immune system, we expect the immune-enhancing activity of cAR due to its compositional similarities to field-cultivated ginseng.

To evaluate the immunomodulatory effect of cAR of ginseng, we also tested the delayed-type hypersensitivity (DTH) response and the carbon clearance assay. These two assays are also classical tests that measure the immune response in vivo. DTH measures the increased volume of each hind footpad as an indicator of enhanced cell-mediated immunity, while the carbon clearance assay determines the phagocytic activities of macrophages. As shown in previous results, the cAR extract was shown to perform significant activities in enhancing the immune system of mice in assays measuring isolated immune cells from mice. However, the cAR extract did not show a positive immunomodulatory effect during these assays that directly monitored the mice. We will further explore the pharmacological behavior of cAR in mice.

The results of the network pharmacology analysis suggested that the main chemical components of cAR, such as the ginsenosides PPT, F1, Rh2, CK, and 20(*S*)-Rg3, potentially act on key targets including TNF, AKT-1, IL-6, VEGFA, and IL-1β. These results are consistent with some previous publications. The ginsenoside Rh2 has been reported for its activities in improving IL-2 production in vitro [50] and increasing the number of T cells in a melanoma mouse model [51]. Rg3 is another ginsenoside that has been well studied for its anti-tumor activity. It was also shown to perform activities in enhancing the phagocytosis in macrophages [52], as well as regulating cytokines and transcription factors [53]. We also identified key signaling pathways, namely the PI3K–Akt pathway, AGE–RAGE signaling pathway, and MAPK signaling pathway, which cAR may affect. These pathways have also been reported as the key targets of ginsenosides [54,55]. This information provides some valuable hypothetical points for further investigation. It is imperative to validate these findings experimentally.

## 4. Materials and Methods

### 4.1. Materials and Reagents

Fresh adventitious roots were provided by Tonghua Herbal Biotechnology, Co., Ltd. (Tonghua, China). The fresh adventitious roots were air-dried, ground, and sieved with a Chinese National Standard Sieve 3 (R40/3 Series). The homogeneous powder obtained was refluxed with 40% ethanol three times (for 2 h, 2 h, and 1 h each time). Then, the extracts were combined, concentrated, and evaporated until their relative density was 1.08 to 1.12 (measured at 70 °C). After that, the concentrated liquid was dried via spray-drying to obtain the final cultured adventitious root sample (cAR). The cAR powder was dissolved in 70% methanol, and after being filtered, the methanolic solution was injected directly into a UPLC system.

The ginsenosides Rf, F2, Ro, Rb1, Rb2, Rb3, Rc, Re, Rg1, 20(*S*)-Rg3, and PPT were provided by the School of Chemistry at Jilin University. The notoginsenosides Fe, Fd, Rd2, and Rg5 were purchased from Chengdu Desite Bio-Technology company (Chengdu, China). The notoginsenosides R2, Rg2, F1, Rd, CK, and F4 were obtained from Chengdu Push Bio-technology Co., Ltd. (Chengdu, China). UPLC-MS-grade methanol and acetonitrile were purchased from Thermo Fisher Scientific Inc. (Waltham, MA, USA), while MS-grade formic acid was purchased from Sigma-Aldrich. Leucine enkephalin and sodium formate were purchased from Waters Technologies Corporation (Milford, MA, USA). Deionized water was obtained from Guangzhou Watson’s Food & Beverage Co., Ltd. (Guangzhou, China). The other chemicals were of analytical grade.

Calf serum (Invitrogen) was bought from Beijing Bioway (Beijing, China), while Hank’s solution was obtained from Beijing Solarbio Sciences & Technology Co., Ltd. (Beijing, China). PBS buffer (pH 7.2–7.4) was purchased from Thermo Fisher (Waltham, MA, USA). Chicken blood red cells, Indian ink, and Na_2_CO_3_ were purchased from Shanghai Yuanye Bio-Technology Co., Ltd. (Shanghai, China). Sheep red blood cells (SRBCs) were bought from Beijing Hancheng Bio-Technology Co., Ltd. (Beijing, China).

The instruments employed in this study included the following: a Waters Xevo G2-S Q-TOF mass spectrometer (Waters Co., Milford, MA, USA), an ACQUITY UPLC, MassLynx™ V4.1 workstation and UNIFI^®^ v1.7 (Waters Technologies Corporation, Milford, MA, USA), an N-A35 nitrogen generator (Shanghai Jinlang Technology Co., Ltd. Shanghai, China), a KQ-250B ultrasonic cleaner (Jiangsu Kunshan Ultrasonic Instrument Corporation, Kunshan, China), a TGL-16aR super speed centrifuge (Shanghai Anting Scientific Instrument Factory, Shanghai, China),a PTX-FA2105 electronic balance (Fujian Huazhi Electronic Technology Co,. Ltd. Fuzhou, China), an Automatic Biochemical Analyzer Chemray 240 (Shenzhen Leadman Biochemistry Co., Ltd. Shenzhen, China), an Epoch microplate reader (BioTek, Santa Clara, CA, USA), a TU-1810PC UV-VIS spectrophotometer (Beijing Puxi Instrument Co., Ltd., Beijing, China), and an Agilent 8453 UV-VIS spectrophotometer (Agilent, Santa Clara, CA, USA).

UPLC-Q/TOF-MS-coupled UNIFI analysis. The chemical ingredients of cAR were determined via UPLC-QTOF-MS-coupled UNIFI analysis. Chromatographic separation was performed using a Waters ACQUITYUPLC BEH C_18_ column (100 mm × 2.1 mm, 1.7 μm, Waters Co., Milford, MA, USA). The temperatures of the UPLC column and autosampler were set to 30 °C and 15 °C, respectively. Mobile phase A consisted of 0.1% formic acid in water (*v*/*v*), while mobile phase B applied 0.1% formic acid in acetonitrile (*v*/*v*). The gradient elution was as follows: 0–2 min, 10% B; 2–26 min, 10% → 100% B; 26–29 min, 100% B; 29–29.1 min 100% → 10% B; and 29.1–32 min, 10% B with a flow rate of 0.4 mL/min. For a weak or strong wash solvent, 10% or 90% acetonitrile/water (*v*/*v*) was used, respectively.

The MS^E^ system working conditions were as follows: electrospray ion source (ESI) temperature: 150 °C; desolvation temperature: 400 °C; desolvation gas flow: 800 L/h; cone voltage: 40 V; and cone gas flow: 50 L/h. The capillary voltages were 2.2 kV for the negative mode. The low-energy function was 6 V, while the high-energy function was 20–40 V. The masses recorded were in the range of 100 to 1500 Da. Leucine enkephalin (5 μL) was injected at a rate of 15 μL/min as an external reference with an *m*/*z* of 554.2615 in the negative mode. Continuum Mode was used to record the MassLynx data.

The components of ginseng cAR were then analyzed. The raw MS data were compressed using the Waters Compression and Archival Tool (v1.10) and screened using the streamlined workflow of the UNIFI 1.7.0 software [44,56]. The minimum peak area was set to 200 for two-dimensional (2D) peak detection, while the peak intensities of low and high energy were set to greater than 1000 and 200 counts, respectively. The negative adducts were -H and +COOH. The mass error was set to ±5 ppm. A self-built database was established by inquiring about the composition of ginseng in PubMed, ChemSpider, Web of Science, and Medline and imported into the Waters Traditional Medicine Library module of UNIFI for MS data analysis. The identified components were further confirmed by comparing the retention times and characteristic MS fragments with published results.

### 4.2. Quantitative Analysis of Total Saponins and Total Polysaccharides

Total saponins. (i) Solution preparation: A standard stock solution of the ginsenoside Re (1 mg/mL) was prepared in methanol. An 8% vanillin solution was prepared by dissolving 0.8 g of vanillin in anhydrous ethanol (10 mL). A 72% sulfuric acid solution was prepared by adding 72 mL of sulfuric acid to an appropriate amount of water, cooling it to room temperature, and diluting it to 100 mL with water. (ii) Preparation of the test solution: An accurately weighed quantity of 1 g cAR was wrapped with neutral filter paper and placed in a Soxhlet extractor. It was then extracted with ether and soaked in methanol overnight. Afterward, it was refluxed with methanol six times, and the methanol was combined, vacuum-evaporated, and dried in a water bath. The resulting extraction was dissolved in water (30 mL to 40 mL) and extracted with water-saturated n-butanol (30 mL) four times. The upper liquid was evaporated to dryness, dissolved, and diluted with methanol to 5.0 mL, creating the test solution. (iii) Drawing the standard curve: 10 μL, 20 μL, 30 μL, 40 μL, 60 μL, 80 μL, and 100 μL of the Re reference solution were accurately measured, respectively, into tubes. The methanol was evaporated in a water bath (<90 °C). Then, 0.5 mL of the 8% vanillin solution and 5 mL of the 72% sulfuric acid solution were added to each tube. The tubes’ contents were mixed well and heated at 60 °C for 10 min; then, they were immediately cooled in ice water for 10 min. The absorbance was measured at 544 nm, the standard curve was drawn, and the cAR content was calculated using the linear regression equation. (iv) Determination of the total saponin content: The steps in (iii) were repeated using the substance being examined. Next, 10 μL to 40 μL of the test solution was accurately measured instead of the reference solutions. The total saponin content was calculated according to Equation (1) (the total saponin content (%); [*CONC*], the mass of the test sample calculated using the regression equation (μg); *V*_1_, the constant volume (mL); *V*_2_, the sample volume (μL); *m*, the weighed mass of the test sample (mg)).

(1) $$X=\frac{\frac{\left[CONC\right]}{V2}\times V1}{m}\times 100$$

Total polysaccharides. (i) Solution preparation: An accurately weighed quantity of 1.0000 g of glucose standard (dried at 105 °C to a constant weight) was dissolved and diluted with distilled water to 100 mL, yielding a standard stock solution of glucose (10 mg/mL). Subsequently, it was further diluted to 0.1 mg/mL. A phenol solution (50 g/L) was prepared by dissolving 5.0 g of phenol in 100 mL of distilled water. (ii) Preparation of the test solution: An accurately weighed quantity of 2.00 g of cAR was extracted with 70 mL of distilled water using ultrasonic for 30 min. It was then extracted for 4 h at 100 °C in a water bath, cooled to room temperature, and diluted to 100 mL. Next, 5 mL of this solution was taken and mixed with 15 mL of an 80% ethanol solution. After centrifuging at 10,000 r/min for 10 min, the supernatant was discarded, and the residual was dissolved in 5 mL of an 80% ethanol solution and centrifuged. The supernatant was again discarded, and the residual was dissolved and diluted to 100 mL with distilled water, serving as the test solution. (iii) Drawing the standard curve: 0 mL, 0.1 mL, 0.2 mL, 0.4 mL, 0.6 mL, 0.8 mL, 1.0 mL, 1.2 mL, 1.4 mL, and 1.6 mL of the glucose reference solution were accurately measured, respectively, into a 25 mL tube with a stopper. The solution was diluted to 2.0 mL with water, and then 1.0 mL of a 5% phenol solution was added. The contents were mixed well, and 5 mL of the sulfuric acid solution was quickly added. The contents were shaken for 5 min, allowed to stand for 10 min, heated in a boiling water bath for 20 min, cooled to room temperature, and measured for absorbance at 486 nm. (iv) Determination of the total polysaccharide content: The operation in (iii) was repeated using the substance being examined. Then, 2 mL of the test solution was accurately measured instead of the reference solutions, and the total polysaccharide content was calculated according to Equation (2) (X, the total polysaccharide content (g/100 g); C1, the mass of the test sample calculated using the regression equation (μg/mL); f, the dilution ratio of the solution; V, the constant volume (mL); m, the weighed mass of the test sample (g)):

(2) $$X=\frac{C1\times f\times V}{m\times 1000\times 1000}$$

### 4.3. Immunomodulatory Activity of Cultured Adventitious Ginseng Root

Animals and groups. Three-week-old male KM breeding mice (body weight: 18–22 g) were purchased from SPF (Beijing) Biotechnology Co., Ltd. (Beijing, China) The mice had an acclimation period of 7 days before they were used in the experiments. Throughout the experiment, the mice had free access to food, and a 12-h light/12-h dark cycle was maintained. The mice were euthanized through cervical dislocation. All animal experiments were approved by the Experimental Animal Ethics Committee of the Academic Committee at Beijing University of Chinese Medicine, Protocol No. BUCM-4-2022092904-3120. The number of mice used is indicated in the main text.

The mice were divided into 5 groups based on the dosage of cAR tested, that is, 0 (the negative control), 21, 42, 83, and 125 mg/kg of body weight (BW) of the mice. Each group contained 10–15 mice. The cAR extract was delivered through oral gavage (10 mL/kg of body weight) once per day for 30 days. For immunization, the mice were administered 0.2 mL of 2% SRBC through intraperitoneal (i.p.) injection from day 25 once per day for 5 days. The mice’s body weights were monitored once per week from the first day of the cAR treatment. The thymus/BW ratio and the spleen/BW ratio of the mice were measured after dosing with cAR for 30 days via oral gavage.

Serum preparation. After 30 days of cAR treatment, the mice’s eyeballs were dissected, and their blood was collected. The blood was centrifuged at 3000 rpm at 4 °C for 15 min to separate the serum for further use.

Spleen cell suspension preparation. The spleen was dissected and placed in Hank’s solution under sterile conditions. The spleen was gently minced using forceps and passed through a 200-mesh sieve or four-layer gauze to obtain a single-cell suspension. The suspension was centrifuged at 1000 rpm for 10 min and further washed twice with Hank’s solution. The spleen cells were then suspended in 1 mL of culture medium. Cell viability was determined with trypan blue staining and was required to be above 95%. The cell concentration was adjusted to 3 × 10^6^ cells/mL. After 5 days of SRBC immunization, a spleen cell suspension of SRBC-immunized mice was prepared following the same procedure as described above, and the concentration was adjusted to 5 × 10^6^ cells/mL in Hank’s solution.

Spleen lymphocyte proliferation. We employed an MTT-based assay to evaluate the proliferation of spleen lymphocyte with modifications [57]. After 30 days of cAR dosing, the mouse spleens were collected, and single-cell suspensions were prepared as described above. Each sample was added to two wells of a 24-well plate (1 mL/well). These wells were further supplied with 75 μL of concanavalin A (Con A) or left blank for the negative control. The plate was incubated under 5% CO_2_ and 37 °C for 72 h. Four hours before the end of the culture, 0.7 mL of the supernatant from each well was gently replaced with 0.7 mL of fetal-bovine-serum-free RPMI1640. Simultaneously, 50 μL of MTT (5 mg/mL) was added to each well. After the completion of the culture, 1 mL of acidic isopropanol was added to each well and mixed gently to completely dissolve the purple crystals. Then, each sample was added to three wells of a 96-well plate to measure the optical density (OD) at a 570 nm wavelength. The OD difference between the samples with and without Con A represented the spleen lymphocyte proliferation.

Quantitative hemolysis of the sheep red blood cell (QHS) assay. The antibody-producing capabilities of splenic cells were assessed using the QHS method described by Simpson with modifications [58]. The mice were administered cAR for 30 days, while SRBC was used for immunization from day 25. The mice were then euthanized through cervical dislocation, and their spleens were dissected to prepare a cell suspension as described above. The splenic cell suspension was mixed with Tris-NH_4_Cl (pH 7.2, 0.017 M Tris, and 0.75% NH_4_Cl) at room temperature for 10 min to lyse the red blood cells and then centrifuged at 2000 rpm for 5 min, and the supernatant was discarded. The cells were washed twice with 5 mL of PBS. The cells were centrifuged at 2000 rpm for 5 min, and the supernatant was discarded. Next, 1 mL of PBS was added for cell counting, and the concentration was adjusted to 2 × 10^7^ cells/mL. Then, 0.2 mL of the spleen cell suspension, 0.2 mL of SRBC, and 0.2 of mL guinea pig blood were added together as the experimental group, while the spleen cell suspension was replaced with 0.2 of mL PBS in the control group. The samples were mixed and incubated at 37 °C for 1 h. Then, they were centrifuged at 3000 rpm for 5 min. The supernatant was added to 96-well plates with 0.1 mL/well for three replicates of each sample. The OD at 413 nm was measured.

Serum hemolysis assay (HC_50_). The serum hemolysis assay was performed based on the description by Jiang with modifications [59]. The mice were administered cAR for 30 days, while SRBC was used for immunization from day 25. The mice were then sacrificed through cervical dislocation, and serum from their eyeballs was prepared as described above. The serum sample was diluted 500-fold with SA buffer. To 50 μL of diluted serum, 25 μL of 10% (*v*/*v*) SRBC and 50 μL of complement (1:8 diluted with SA) were added for the experimental group, while the serum was replaced with an SA buffer in the control group. The samples were incubated at 37 °C for 30 min. The reactions were terminated by placing the plate on ice. The samples were centrifuged at 1500 rpm for 10 min. Then, 50 μL of the sample supernatant was transferred to a 96-well plate, and 150 μL of hemoglobin oxidizing agent was added. For the 50% hemolysis sample, 12.5 μL of 10% (*v*/*v*) SRBC was used, and a hemoglobin oxidizing agent was added to a total volume of 200 μL. It was mixed thoroughly, and the samples were left to sit for 10 min. The OD at 540 nm was measured. HC_50_ was calculated as follows (Equation (3)):

(3) $${HC}_{50}=\frac{sample\;OD}{50\%\;hemolysis\;OD}\times dilution\;ratio$$

The phagocytic function of peritoneal macrophages. The phagocytic function of the peritoneal macrophages was measured according to the method described by Okimura with modifications [60]. The mice were administered cAR for 30 days, and 0.2 mL of SRBC (2%) was used for immunization through i.p. injection from day 25. The mice were then euthanized through cervical dislocation, and 4 mL of calf serum containing Hank’s solution was injected into the peritoneal cavity. The abdomen was gently massaged 20 times to thoroughly wash out the peritoneal macrophages. Next, 2 mL of peritoneal lavage fluid was collected from a small incision in the abdominal wall. Then, 1 mL of peritoneal fluid was mixed with 0.5 mL of chicken red blood cells (1% CRBC). A 0.5 mL mixture was added within the gelatin ring on a glass slide and incubated at 37 °C for 15–20 min. After incubation, the non-adherent cells were quickly rinsed off with saline. The adherent cells were fixed in methanol for 1 min and stained with Giemsa solution for 15 min. The slide was then washed with distilled water and air-dried. The number of macrophages was counted (100 per slide). The phagocytosis rates were calculated to evaluate the phagocytic function. The phagocytosis rate (Equation (4)) is the percentage of macrophages that engulfed CRBCs out of every 100 macrophages.

(4) $$phagocytosis\;rate=\frac{macrophages\;engulfed\;CRBC}{100\;macrophages}\times 100\%$$

Natural killer cell activity. The activities of natural killer cells were evaluated according to the method described by Lv [61]. The mice were treated with cAR for 30 days via oral gavage. The mice were sacrificed through cervical dislocation on day 31, their spleens were collected, and a single-cell suspension was prepared as described above. The cell concentration was adjusted to 2 × 10^7^ cells/mL. Then, 100 μL of effector and target cells (50:1) were added to a U-shaped, 96-well cell culture plate. A culture medium was used in place of effector cells for the natural release group, while 1% NP40 or 2.5% Triton was used in place of effector cells for the maximum release group. Three replicate experiments were conducted for each group. The plates were incubated at 37 °C with 5% CO_2_ for 4 h. After centrifuging at 1500 rpm for 5 min, 100 µL of the supernatant was transferred to a flat 96-well plate, and 100 μL of LDH was added for 3–10 min incubation at room temperature. The reaction was stopped by adding 1 M of HCl (30 μL/well). The OD was measured at a 490 nm wavelength. The NK cell activity was calculated as follows (Equation (5)):

(5) $$NK\;activity=\frac{Experimental-spontaneous\;\;release\;}{maximum-spontaneous\;\;release}\times 100\%$$

Statistical analysis. All data were analyzed with SPSS 2.0. Statistical significance was assessed using a one-way ANOVA. The least significant difference (LSD) was used for the homogeneity of variances, while Tamhane’s T2 was used for the heterogeneity of variances. For non-normal distributed data, the Kruskal–Wallis test was used. The values are presented as means ± SDs, and *p* < 0.05 was considered statistically significant.

### 4.4. Network Pharmacology Analysis

The components of ginseng cAR determined via UPLC-MS were further analyzed through network pharmacology analysis to explain the underlying mechanism of their immunomodulatory effect. The key components, related proteins, and pathways were analyzed using Cytoscape 3.10.0 (Cytoscape Consortium, San Diego, CA, USA). SwissTargetPrediction and Symmap were employed to predict the protein target of the cAR components. The protein targets related to immunodeficiency were identified through Malacards and DisGeNET using the keywords ‘Immunodeficiency disease’, ‘Acquired Immunodeficiency Syndrome’, or ‘Immune System Disease’. (1) For ‘key protein targets’, the cAR protein target and immunodeficiency-related proteins were cross-compared, and the intersection targets formed the ‘core targets’. A protein–protein interaction (PPI) network was then generated by importing the ‘core targets’ list into the String database (http://string-db.org/cgi/input/pl, accessed on 1 March 2023 to 25 May 2023). The highest confidence was set to 0.4, and the discrete nodes were selected ‘hiding’. The ‘key protein targets’ with the highest degree values were identified by analyzing the PPI network through Cytoscape 3.10.0. (2) For the ‘key cAR components’, all 60 cAR components and 121 ‘core targets’ were considered in constructing a ‘components-core targets’ network through Cytoscape 3.10.0. The key components were identified based on the degree values. (3) For the ‘key signaling pathway’, the ‘core targets’ were analyzed for gene ontology (GO) enrichment via Gprofiler (https://biit.cs.ut.ee/gpprofiler/convert, accessed on 1 March 2023 to 25 May 2023) and Kyoto Encyclopedia of Genes and Genomes (KEGG) pathway enrichment analysis through DAVID (https://david.ncifcrf.gov/, accessed on 1 March 2023 to 25 May 2023). Omicshare (https://auth.lifemapsc.com, accessed on 1 March 2023 to 25 May 2023) was employed for data visualization. The top-10-ranked enriched signaling pathways were used to construct a ‘components-targets-pathways’ network.

## 5. Conclusions

Ginseng has been widely used and studied due to its highly valuable pharmacological potency. However, it is essential to increase the production of ginsenosides, which are the main biologically active components in ginseng. The culturing of adventitious ginseng root in vitro has shown great potential. In this study, we identified 60 ingredients from the cAR of ginseng extracts using UPLC-QTOF-MS. The extracts showed positive results in improving spleen lymphocyte proliferation, enhancing the hemolysis actions of splenic cells and serum, promoting the phagocytic function of macrophages, and increasing NK cell activities. Network pharmacology analysis suggested that the immunomodulatory effect of cAR may work through (1) protein targets including TNF, AKT1, IL-6, VEGFA, and IL-1β, (2) ginsenosides including PPT, F1, Rh2, CK, and 20(*S*)-Rg3, or (3) the PI3K–Akt, AGE–RAGE, and MAPK signaling pathways.

## Figures and Tables

**Table 1:** Compounds identified from ginseng cAR extract via UPLC-QTOF-MS^E^.

No.	*t_R_* (min)	Formula	Theoretical Mass (Da)	Calculated Mass (Da)	Mass Error (ppm)	MS^E^ Fragmentation	Identification	Reference
1	0.59	C_12_H_22_O_11_	342.1162	342.1155	–2.15	387.1137 [M+HCOO]^−^, 341.1084 [M−H]^−^, 179.0562 [M−H−Glu]^−^	Sucrose	s
2	0.65	C_7_H_12_O_6_	192.0634	192.0656	0.65	191.0584 [M−H]^−^, 173.0460 [M−H−H_2_O]^−^, 127.0407 [M−H−H_2_O−HCOOH]^−^	Quinic acid	s
3	1.68	C_11_H_12_N_2_O_2_	204.0899	204.0905	3.04	203.0832 [M−H]^−^, 159.0938 [M−H−CO_2_]^−^	Tryptophan	s
4	2.50	C_16_H_18_O_9_	354.0951	354.0944	−1.93	353.0871 [M−H]^−^, 191.0562 [M−H−C_9_H_6_O_3_]^−^, 173.0463 [M−H−C_9_H_8_O_4_]^−^	1-*O*-caffeoylquinic acid	[31]
5	4.31	C_16_H_18_O_8_	338.1002	338.0992	−2.95	337.0920 [M−H]^−^, 191.0559 [M−H−C_9_H_6_O_2_]^−^, 145.0300 [M−H−C_7_H_12_O_6_]^−^	3-*O*-*p*-Coumaroylquinic acid	[32]
6	5.02	C_17_H_20_O_9_	368.1107	368.1098	–2.44	367.1026 [M−H]^−^, 179.0356 [M−H−C_8_H_12_O_5_]^−^, 135.0455 [M−H−C_9_H_12_O_7_]^−^	Methyl 4-caffeoylquinate	[33]
7	5.94	C_48_H_82_O_19_	962.5450	962.5436	–1.50	1007.5418 [M+HCOO]^−^, 961.5354 [M−H]^−^, 799.4800 [M−H−Glu]^−^	Notoginsenoside N	[34]
8	5.94	C_48_H_82_O_19_	962.5450	962.5436	−1.43	1007.5418 [M+HCOO]^−^, 781.4742 [M−H−Glu]^−^	Majoroside F6	[35]
9	6.61	C_30_H_54_O_5_	494.3971	494.3958	−2.49	539.3940 [M+HCOO]^−^, 347.2520 [M−C_8_H_19_O_2_]^−^	Dammar-3*β*, 6*α*, 12*β*, 20R, 25-pentaol	CFM-ID
10	7.26	C_48_H_82_O_19_	962.5450	962.5428	−2.25	961.5325[M−H]^−^, 621.4401[M−H−H_2_O−2Glu]^−^	Notoginsenoside R6	[36]
11	7.72	C_48_H_82_O_19_	962.5450	962.5414	−3.62	1007.5393 [M+HCOO]^−^, 799.4836 [M−H−Glu]^−^	20-*O*-D-glucopyranosyl-ginsenoside Rf	[37]
12	7.74	C_47_H_80_O_18_	932.5345	932.5318	–2.84	977.5300 [M+HCOO]^−^, 799.4836 [M−H−Xyl]^−^	Notoginsenoside R1	s
13	8.17	C_42_H_72_O_14_	800.4922	800.4882	–5.04	845.4864 [M+HCOO]^−^, 799.6775 [M−H]^−^, 637.4304 [M−H−Glu]^−^, 476.3833 [M−H−2Glu]^−^	Ginsenoside Rg1	s
14	8.21	C_48_H_82_O_18_	946.5501	946.5454	–4.98	991.5436 [M+HCOO]^−^, 945.5383 [M−H]^−^, 783.4896 [M−H−Glu]^−^, 476.3833 [M−H−2Glu−Rha]^−^	Ginsenoside Re	s
15	8.52	C_44_H_74_O_15_	842.5028	842.5006	−2.58	841.4933 [M−H]^−^, 799.4831 [M−Ac]^−^, 679.4391 [M−Rha]^−^, 637.4319 [M−Ac−Rha]^−^	Vinaginsenoside R1	[38]
16	8.80	C_51_H_84_O_21_	1032.5505	1032.5474	–3.00	1031.5401 [M−H]^−^, 987.5516 [M−H−Ac]^−^	Malonyl-ginsenoside Re	[39]
17	9.20	C_48_H_82_O_19_	962.5450	962.5419	–3.25	1007.5401 [M+HCOO]^−^, 961.5376 [M−H]^−^, 799.4810 [M−H−Glc]^−^	Ginsenoside Re3	[40]
18	9.41	C_48_H_82_O_19_	962.5450	962.5426	–2.57	1007.5408 [M+HCOO]^−^, 961.5353 [M−H]^−^, 946.5444 [M−H−CH_3_]^−^, 800.4871 [M−H−Glu]^−^	Majoroside F1	[34]
19	10.23	C_42_H_72_O_14_	800.4922	800.4875	–5.83	845.4857 [M+HCOO]^−^, 799.4807 [M−H]^−^, 637.4313 [M−H−Glu]^−^, 475.3780 [M−H−2Glu]^−^	Ginsenoside Rf	s
20	10.51	C_41_H_70_O_13_	770.4816	770.4783	–4.38	815.4765 [M+HCOO]^−^, 769.4706 [M−H]^−^, 637.4313 [M−H−Ara]^−^	Ginsenoside F5	s
21	10.59	C_54_H_92_O_23_	1108.6029	1108.5960	–6.22	1153.5942 [M+HCOO]^−^, 1107.5890 [M−H]^−^, 945.5387 [M−H−Glu]^−^, 765.4771 [M−H−2Glu]^−^	Ginsenoside Rb1	s
22	10.69	C_57_H_94_O_26_	1194.6033	1194.5973	–5.05	1193.5900 [M−H]^−^, 1089.5804 [M−H−mal]^−^, 927.5304 [M−H−mal−Glu]^−^	Malonyl-ginsenoside Rb1	[34]
23	10.72	C_41_H_70_O_13_	770.4816	770.4791	–3.34	815.4773 [M+HCOO]^−^, 769.4724 [M−H]^−^, 637.4290 [M−H−Ara]^−^, 475.3764 [M−H−Ara−Glu]^−^	Notoginsenoside R2	s
24	10.79	C_53_H_90_O_22_	1078.5924	1078.5870	–4.96	1123.5852 [M+HCOO]^−^, 915.5283 [M−H−Glu]^−^, 765.4773 [M−H−Glu−Ara]^−^, 621.4376 [M−H−2Glu−Ara]^−^	Ginsenoside Rb2	s
25	10.82	C_42_H_72_O_13_	784.4973	784.4936	–4.77	829.4918 [M+HCOO]^−^, 637.4306 [M−H−Rha]^−^, 475.3793 [M−H−Glu−Rha]^−^	20(*R*)-Ginsenoside Rg2	s
26	10.86	C_36_H_62_O_9_	638.4394	638.4374	–3.07	683.4356 [M+HCOO]^−^, 637.4293 [M−H]^−^, 475.3793 [M−H−Glu]^−^	20(*S*)-Ginsenoside Rh1	s
27	10.91	C_56_H_92_O_25_	1164.5928	1164.5875	–4.53	1163.5802 [M−H]^−^, 1119.5900 [M−H−CO_2_]^−^, 1059.5708 [M−H−Mal]^−^, 1031.5398 [M−H−Ara]^−^, 945.5390 [M−H−Ara−Mal]^−^	Malonyl-ginsenoside Rc	[34]
28	10.97	C_48_H_76_O_19_	956.4981	956.4938	–4.49	955.4865 [M−H]^−^, 793.4361 [M−H−Glc]^−^	Ginsenoside Ro	s
29	11.03	C_53_H_90_O_22_	1078.5924	1078.5856	–6.26	1123.5852 [M+HCOO]^−^, 1077.5802 [M−H]^−^, 915.5301 [M−H−Glu]^−^, 783.4883[M−H−Glu−Xyl]^−^, 621.4378 [M−H−2Glu−Xyl]^−^	Ginsenoside Rb3	s
30	11.20	C_56_H_92_O_25_	1164.5928	1164.5875	–4.53	1163.5802 [M−H]^−^, 1060.4652 [M−H−Mal]^−^, 928.5332 [M−H−Ara−Mal]^−^, 619.4217 [M−H−2Glu−Ara−Mal]^−^	Malonyl-ginsenoside Rb2	[34]
31	11.29	C_47_H_74_O_18_	926.4875	926.4863	−1.34	925.4790 [M−H]^−^, 569.3833 [M−H−Ara−Glu−HCOOH]^−^	Chikusetsu saponin I_b_	[41]
32	11.45	C_48_H_82_O_17_	930.5552	930.5524	–3.02	873.4846 [M+HCOO]^−^, 784.4798 [M−H−COCH_3_]^−^, 695.2912 [M−H−Xyl]^−^, 491.4938 [M−H−Xyl−Glu−Ac]^−^, 455.2535 [M−H−Xyl−Glu−Ac−2H_2_O]^−^	Gypenoside XI	[34]
33	11.49	C_55_H_92_O_23_	1120.6029	1120.5987	–3.75	1119.5915 [M−H]^−^, 1077.5815 [M−H−Ac]^−^, 915.5313 [M−H−Ac−Glu]^−^, 781.4724 [M−H−Ac−Ara−Glu]^−^	Ginsenoside Rs1	s
34	11.49	C_36_H_62_O_9_	638.4394	638.4381	–2.01	683.4363 [M+HCOO]^−^, 637.4310 [M−H]^−^, 475.3796 [M−H−Glu]^−^	Ginsenoside F1	s
35	11.50	C_53_H_90_O_22_	1078.5924	1078.5896	–2.55	1123.5878 [M+HCOO]^−^, 1077.5646 [M−H]^−^, 915.5313 [M−H−Glu]^−^, 781.4724 [M−H−Ara−Glu]^−^, 576.4474 [M−H−Ara−2Glu]^−^	Ginsenoside Rc	s
36	11.65	C_48_H_82_O_18_	946.5501	946.5448	–5.65	991.5430 [M+HCOO]^−^, 945.5379 [M−H]^−^, 783.4878 [M−H−Glu]^−^, 621.4353 [M−H−2Glu]^−^	Ginsenoside Rd	s
37	11.76	C_51_H_84_O_21_	1032.5505	1032.5457	–4.65	1031.5384 [M−H]^−^, 987.5494 [M−H−CO_2_]^−^, 927.5294 [M−H−mal]^−^, 765.4797 [M−H−mal−Glu]^−^	Malonyl-ginsenoside Rd	[34]
38	11.86	C_55_H_92_O_23_	1120.6029	1120.5991	–3.45	1165.5973 [M+HCOO]^−^, 1159.5869 [M−H]^−^, 985.5371 [M−H−Ara]^−^, 915.5299 [M−H−Glu−Ac]^−^	Ginsenoside Rs2	s
39	12.08	C_48_H_82_O_18_	946.5501	946.5456	–4.81	991.5438 [M+HCOO]^−^, 945.5389 [M−H]^−^, 783.4880 [M−H−Glu]^−^, 621.4338 [M−H−2Glu]^−^	Gypenoside XVII	s
40	12.25	C_47_H_80_O_17_	916.5396	916.5367	–3.16	961.5349 [M+HCOO]^−^, 915.5290 [M−H]^−^, 783.4881 [M−H−Ara]^−^	Notoginsenoside Fe	[34]
41	12.43	C_47_H_80_O_17_	916.5396	916.5342	–5.87	961.5324 [M+HCOO]^−^, 915.5276 [M−H]^−^, 783.4874 [M−H−Ara]^−^, 621.4362 [M−H−Ara−Glu]^−^	Ginsenoside Rd2	s
42	12.59	C_47_H_80_O_17_	916.5396	916.5356	–4.28	961.5338 [M+HCOO]^−^, 915.5283 [M−H]^−^, 621.4359 [M−H−Glu−Ara]^−^	Notoginsenoside Fd	s
43	12.70	C_47_H_80_O_17_	916.5396	916.5343	−5.48	961.7556 [M+HCOO]^−^, 915.7454 [M−H]^−^, 765.4754 [M−H−Xyl]^−^	Chikusetsu saponin Ⅲ	[42]
44	13.34	C_48_H_82_O_17_	930.5552	930.5524	−2.80	975.5507 [M+HCOO]^−^, 765.4807 [M−H−Rha]^−^	Gypenoside X	[43]
45	13.56	C_42_H_72_O_13_	784.4973	784.4933	–5.10	829.4915 [M+HCOO]^−^, 783.4892 [M−H]^−^, 621.4357 [M−H−Glu]^−^, 459.4833 [M−H−2Glu]^−^	Ginsenoside F2	[34]
46	14.05	C_42_H_66_O_14_	794.4453	794.4407	–5.74	839.4409 [M+HCOO]^−^, 795.4334 [M−H]^−^, 613.3729 [M−H−Glu]^−^, 569.3844 [M−H−Glu−H_2_O−Ac]^−^	Chikusetsusaponin IVA	[34]
47	14.52	C_42_H_72_O_13_	784.4973	784.4927	–5.85	829.4909 [M+HCOO]^−^, 783.4880 [M−H]^−^, 621.4374 [M−H−Glc]^−^	20(S)-Ginsenoside Rg3	s
48	14.60	C_42_H_70_O_12_	766.4867	766.4840	–3.58	811.4822 [M+HCOO]^−^, 765.4780 [M−H]^−^, 747.4664 [M+H−H_2_O]^−^, 619.4188 [M−H−Rha]^−^	Ginsenoside F4	s
49	14.67	C_42_H_72_O_13_	784.4973	784.4945	–3.62	829.4921 [M+HCOO]^−^, 783.4902 [M−H]^−^, 621.4396 [M−H−Glu]^−^	20(*R*)-Ginsenoside Rg3	s
50	15.01	C_41_H_64_O_13_	764.4347	764.4325	−2.93	763.4252 [M−H]^−^, 613.3720 [M−H−Xyl]^−^, 569.3848 [M−H−Xyl−CO_2_]^−^	Pseudoginsenoside Rp1	CFM-ID
51	15.28	C_41_H_70_O_12_	754.4867	754.4847	−2.50	799.4829 [M+HCOO]^−^, 621.4355 [M+H−H_2_O−Xyl]^−^,	Chikusetsusaponin Ia	CFM-ID
52	16.27	C_30_H_52_O_4_	476.3865	476.3860	−0.89	521.3843 [M+HCOO]^−^, 475.3791 [M−H]^−^	20(*S*)-Protopanaxatriol	s
53	16.75	C_36_H_62_O_8_	622.4445	622.4425	–3.22	667.4407 [M+HCOO]^−^, 621.4346 [M−H]^−^, 459.3818 [M−H−Glu]^−^	Ginsenoside Rh2	s
54	16.82	C_42_H_70_O_12_	766.4867	766.4854	−1.69	811.4837 [M+HCOO]^−^, 765.6676 [M−H]^−^	Ginsenoside Rg4	[44]
55	17.05	C_42_H_70_O_12_	766.4867	766.4855	–1.64	811.4837 [M+HCOO]^−^, 765.4777 [M−H]^−^, 603.4244 [M−H−Glu]^−^	Ginsenoside Rg5	[45]
56	17.35	C_36_H_62_O_8_	622.4445	622.4431	–2.13	667.4413 [M+HCOO]^−^, 621.4354 [M−H]^−^, 459.3826 [M−H−Glu]^−^	Ginsenoside CK	s
57	21.34	C_18_H_30_O_2_	278.2246	278.2244	–0.82	277.2244 [M−H]^−^, 259.2146 [M−H−H_2_O]^−^, 135.1178 [M−H−C_8_H_14_O_2_]^−^	Linolenic acid	s
58	22.80	C_18_H_32_O_2_	280.2402	280.2398	–1.54	325.2387 [M+HCOO]^−^, 279.2325 [M−H]^−^, 261.2225 [M−H−H_2_O]^−^	Linoleic acid	s
59	24.49	C_18_H_34_O_2_	282.2559	282.2552	–2.34	327.2541 [M+HCOO]^−^, 281.2479 [M−H]^−^, 236.2496 [M−H−COOH]	9-Octadecenoic acid	s
60	27.95	C_35_H_60_O_6_	576.4390	576.4377	−2.03	621.4359 [M+HCOO]^−^, 575.3045 [M−H]^−^	*β*-daucosterol	CFM-ID

s: identified with the standard. CFM-ID: compared with CFM-ID 4.0.

**Table 2:** Results of five assays evaluating the immunomodulatory effect of cAR extracts, including spleen lymphocyte proliferation, the quantitative hemolysis of SRBC (QHS), a hemolysis assay, the phagocytic function of the peritoneal macrophages, and natural killer cell activities. The results are presented as means ± SDs.

	Lymphocyte Proliferation	QHS Assay	Hemolysis Assay	Phagocytic Function	NK Cell Activity
	Stimulation Index	OD	HC_50_ U/mL	Phagocytosis Rate %	Cell Activity %
Control	0.231 ± 0.038	0.297 ± 0.010	56.031 ± 5.465	25.30 ± 1.64	11.94 ± 3.92
21 mg/kg BW	0.396 ± 0.023	0.346 ± 0.007	71.531 ± 13.578	33.20 ± 2.90	35.29 ± 12.25
42 mg/kg BW	0.358 ± 0.033	0.345 ± 0.008	81.651 ± 21.554	42.40 ± 4.22	39.64 ± 3.65
83 mg/kg BW	0.317 ± 0.037	0.325 ± 0.005	91.397 ± 48.498	48.90 ± 3.07	50.18 ± 1.49
125 mg/kg BW	0.336 ± 0.050	0.387 ± 0.032	91.248 ± 40.844	72.00 ± 3.92	38.89 ± 6.59

**Table 3:** Top-10-ranking cAR components with the highest degree values.

No.	Compound Name	Degree
1	20(*S*)-Protopanaxatriol	37
2	Ginsenoside F1	35
3	Ginsenoside Rh2	34
4	Ginsenoside CK	32
5	20(*S*)-Ginsenoside Rg3	25
6	Ginsenoside Rg5	16
7	20(*E*)-Ginsenoside F4	12
8	Ginsenoside Rg4	12
9	20(*S*)-Ginsenoside Rh1	12
10	Ginsenoside Rg1	12

## Data Availability

Data are contained within the article and Supplementary Materials.

## Supplementary Materials

The following supporting information can be downloaded at: https://www.mdpi.com/article/10.3390/molecules29010111/s1, Figure S1: Chemical structures of compounds identified in cAR of ginseng extract; Table S1: Body weights and organ/body weight ratio of mice in each group. Results are in average ± SD. Body weight is measured in gram.
