# Supplementary material for: A Qualitative Analysis of Cultured Adventitious Ginseng Root’s Chemical Composition and Immunomodulatory Effects
Source: Molecules. 2023 Dec 23;29(1):111. doi: 10.3390/molecules29010111 (PMC10780104; doi:10.3390/molecules29010111)
Supplement: Supplementary file 1 [file molecules-29-00111-s001.zip › molecules-2751002-supplementary.pdf]

Supplementary information

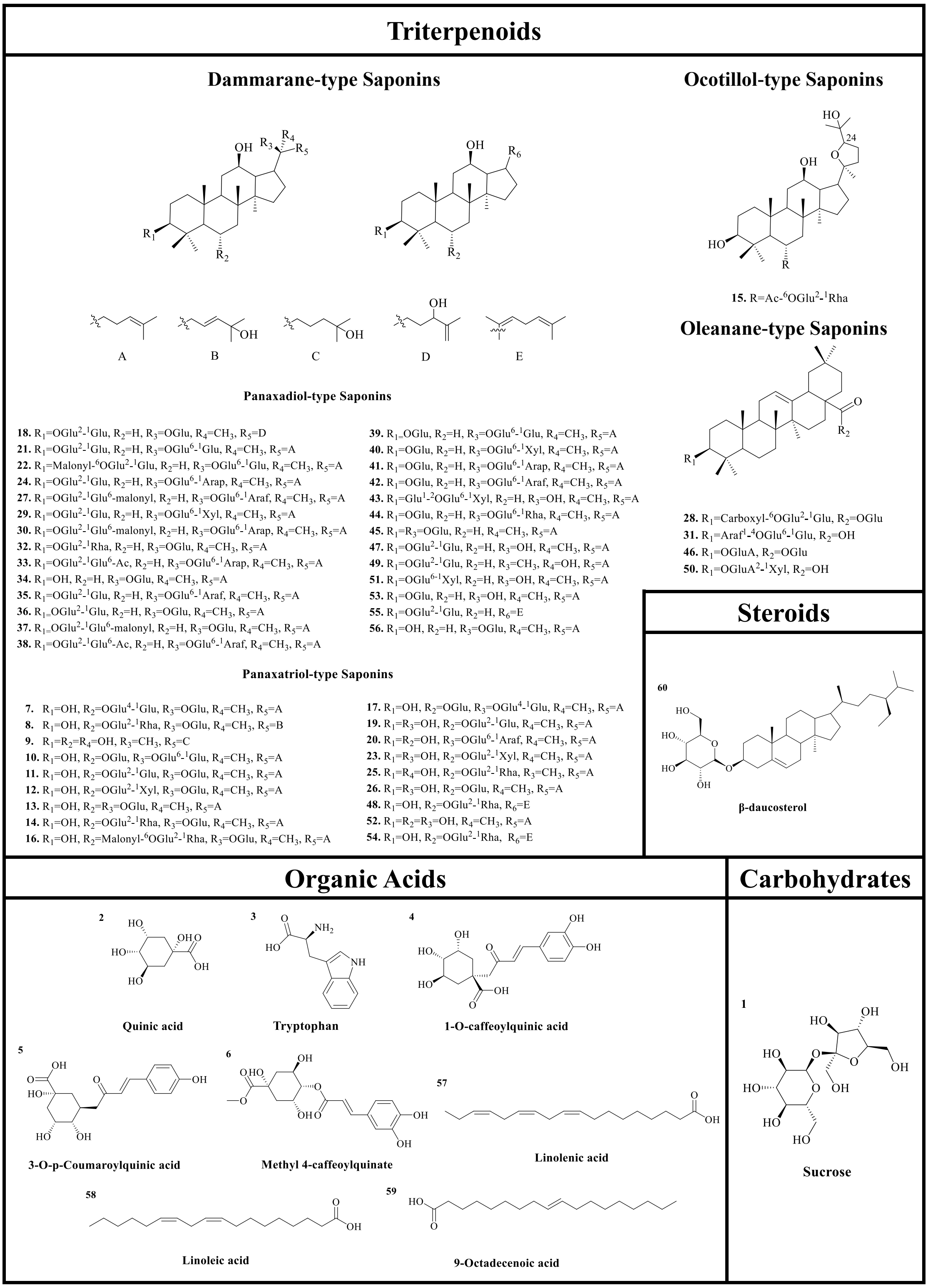

Figure S1 Chemical structures of compounds identified in cAR of ginseng extract.

Table S1 Body weights and organ / body weight ratio of mice in each group. Results are in average±SD. Body weight is measured in gram.

	Body weight (week)					Organ / BW	
	0	1	2	3	4	Thymus/BW	Spleen/BW
Control	30.85±1.67	38.25±2.79	41.50±3.27	44.29±3.33	46.83±3.65	0.00165±0.00017	0.00287±0.00047
21 mg/Kg BW	31.13±1.74	38.11±3.28	41.53±3.84	43.87±3.46	45.77±3.79	0.00142±0.00028	0.00245±0.00028
42 mg/Kg BW	31.31±1.40	37.40±2.43	41.74±2.86	43.89±3.82	45.96±4.02	0.00149±0.00033	0.00266±0.00043
83 mg/Kg BW	30.93±1.61	38.32±2.99	42.88±3.44	44.62±3.36	46.6±3.69	0.00156±0.00045	0.00285±0.00067
125 mg/Kg BW	30.79±1.50	37.20±2.47	41.02±3.02	43.07±3.32	45.37±3.28	0.00143±0.00024	0.00284±0.00055
